# Supplementary material for: Competing Vegetation Structure Indices for Estimating Spatial Constrains in Carabid Abundance Patterns in Chinese Grasslands Reveal Complex Scale and Habitat Patterns
Source: Insects. 2020 Apr 16;11(4):249. doi: 10.3390/insects11040249 (PMC7240609; doi:10.3390/insects11040249)
Supplement: Supplementary file 1 [file insects-11-00249-s001.pdf]

Competing vegetation structure indices for estimating spatial constrains in carabid abundance patterns in Chinese grasslands reveal complex scale and habitat patterns

Noelline Tsafack [1,*], Simone Fattorini [2,*], Camila Benavides Frias [3], Yingzhong Xie [1], Xinpu Wang [1], and François Rebaudo [3]

[1] School of Agriculture, Ningxia University, 489 Helanshan West Road, 750021 Yinchuan, Ningxia, P.R. China; noellinetsafack@gmail.com (N.T.); xieyz@nxu.edu.cn (Y.X.); wangxinpu@nxu.edu.cn (X.W.)
[2] Department of Life, Health, and Environmental Sciences, University of L'Aquila, 67100, L'Aquila, Italy; simone.fattorini@univaq.it
[3] French National Centre for Scientific Research, UMR EGCE, IRD, CNRS, Paris-Saclay University, 91190 Gif-sur-Yvette, France; alimac.cherry@gmail.com (C.B.F.); francois.rebaudo@ird.fr (F.R.);
* Correspondence: noelline.tsafack@gmail.com (N.T.); simone.fattorini@univaq.it (S.F.)

Received: date; Accepted: date; Published: date

Supplementary Materials:

Table S1. Vegetation indices computed from Landsat8 OLI images from the RStoolbox documentation under GPL (≥3) licence (https://cran.r-project.org/web/licenses/GPL-3).

Index	Description	Bands	Formula
CTVI	Corrected Transformed Vegetation Index	red, nir	(NDVI + 0.5)/sqrt(abs(NDVI + 0.5))
DVI	Difference Vegetation Index	red, nir	s * nir - red
GEMI	Global Environmental Monitoring Index	red, nir	(((nir^2 - red^2) * 2 + (nir * 1.5) + (red * 0.5))/(nir + red + 0.5)) * (1 - ((((nir^2 - red^2) * 2 + (nir * 1.5) + (red * 0.5))/(nir + red + 0.5)) * 0.25)) - ((red - 0.125)/(1 - red))
GNDVI	Green Normalized Difference Vegetation Index	green, nir	(nir - green)/(nir + green)
MNDWI	Modified Normalized Difference Water Index	green, swir2	(green - swir2)/(green + swir2)
MSAVI	Modified Soil Adjusted Vegetation Index	red, nir	nir + 0.5 - (0.5 * sqrt((2 * nir + 1)^2 - 8 * (nir - (2 * red))))
MSAVI2	Modified Soil Adjusted Vegetation Index 2	red, nir	(2 * (nir + 1) - sqrt((2 * nir + 1)^2 - 8 * (nir - red)))/2
NBRI	Normalized Burn Ratio Index	nir, swir3	(nir - swir3)/(nir + swir3)
NDVI	Normalized Difference Vegetation Index	red, nir	(nir - red)/(nir + red)
NDWI	Normalized Difference Water Index	green, nir	(green - nir)/(green + nir)
NDWI2	Normalized Difference Water Index	nir, swir2	(nir - swir2)/(nir + swir2)
NRVI	Normalized Ratio Vegetation Index	red, nir	(red/nir - 1)/(red/nir + 1)
RVI	Ratio Vegetation Index	red, nir	red/nir
SATVI	Soil Adjusted Total Vegetation Index	red, swir2, swir3	(swir2 - red)/(swir2 + red + L) * (1 + L) - (swir3/2)
SAVI	Soil Adjusted Vegetation Index	red, nir	(nir - red) * (1 + L)/(nir + red + L)
SLAVI	Specific Leaf Area Vegetation Index	red, nir, swir2	nir/(red + swir2)
SR	Simple Ratio Vegetation Index	red, nir	nir/red
TVI	Transformed Vegetation Index	red, nir	sqrt((nir - red)/(nir + red) + 0.5)
TTVI	Thiam's Transformed Vegetation Index	red, nir	sqrt(abs((nir - red)/(nir + red) + 0.5))
WDVI	Weighted Difference Vegetation Index	red, nir	nir - s * red
